# Folate and Borneol Modified Bifunctional Nanoparticles for Enhanced Oral Absorption

**DOI:** 10.3390/pharmaceutics10030146

**Published:** 2018-09-04

**Authors:** Yifan Yang, Yunzhi Yin, Jun Zhang, Tiantian Zuo, Xiao Liang, Jing Li, Qi Shen

**Affiliations:** School of Pharmacy, Shanghai Jiao Tong University, 800 Dongchuan Road, Shanghai 200240, China; yfyang20@sjtu.edu.cn (Y.Ya.); yinyunzhi@163.com (Y.Yi.); junzhang007@sjtu.edu.cn (J.Z.); zuottsdhm@sjtu.edu.cn (T.Z.); cpuxiao@126.com (X.L.); dodojing_1@163.com (J.L.)

**Keywords:** docetaxel, nanoparticles, dual-functional, folic acid, borneol, oral absorption

## Abstract

Oral delivery is considered the preferred route of administration due to its convenience and favorable compliance. Here, docetaxel (DTX) loaded polylactic-co-glycolic acid (PLGA) nanoparticles, coated with polyethyleneimine–folic acid (PEI-FA) and polyethyleneimine–borneol (PEI-BO), were designed to enhance oral absorption (FA/BO-PLGA-NPs). The FA/BO-PLGA-NPs were spherical and smooth with an average size of (137.0 ± 2.1) nm. Encapsulation efficiency (EE%) and drug loading (DL%) were (80.3 ± 1.8)% and (2.3 ± 0.3)%, respectively. In vitro release studies showed that approximately 62.1% of DTX was released from FA/BO-PLGA-NPs in media at pH 7.4. The reverted gut sac method showed that the absorption of FA/BO-PLGA-NPs in the intestines was approximately 6.0 times that of DTX. Moreover, cellular uptake suggested that the obtained FA/BO-PLGA-NPs could be efficiently internalized into Caco-2 cells via FA-mediated active targeting and BO-mediated P-glycoprotein (P-gp) inhibition. Pharmacokinetics study demonstrated that after oral administration of DTX at a dose of 10 mg/kg in FA/BO-PLGA-NPs, the bioavailability of FA/BO-PLGA-NPs was enhanced by approximately 6.8-fold compared with that of DTX suspension. FA/BO-PLGA-NPs caused no obvious irritation to the intestines. Overall, the FA/BO-PLGA-NP formulation remarkably improved the oral bioavailability of DTX and exhibited a promising perspective in oral drug delivery.

## 1. Introduction

At present, cancer is one of the leading causes of death in humans [1]. Chemotherapy is an important part of the treatment of cancer, and most of the anticancer drugs used in clinical practice are mainly injected. Oral administration is the most convenient and popular route to treat and increase patient compliance [2,3]; however, low oral bioavailability has become the major challenge in this route. Approximately 70% of drugs exhibit unsatisfactory oral administration due to poor solubility or absorption problems [4,5]. Therefore, the improvement of the oral bioavailability of drugs is the key problem to be tackled.

Docetaxel (DTX) is suitable for the treatment of locally advanced nonsmall cell lung cancer or metastatic breast cancer [6]. However, due to its poor solubility, the clinical application of DTX is injection, which has many side effects [7]. In order to reduce the side effects and improve the efficacy of DTX, many studies have been designed, such as using nanoparticles, liposomes, and micelles, among which the oral formulations have the advantages of commercial forms [8,9,10,11]. The polylactic-co-glycolic acid (PLGA) polymer has been approved by the FDA and is extensively used in drug delivery because of its good biodegradability and biocompatibility. Especially, PLGA is reported to improve bioavailability in oral absorption, which is probably attributed to its increased drug solubility, reduced first-pass metabolism and its promotion of preferential absorption in gastrointestinal tract [12]. However, the weak negative charge of PLGA nanoparticles often leads to low bioavailability, which is caused by limits on their ability to interact with negatively charged plasmids and intracellular uptake [13]. To modify the surface charge of the PLGA nanoparticles, the cationic polymer polyethyleneimine (PEI) might be a potential candidate material. Positively charged PEI can improve the nanoparticle absorption through the electrostatic interaction of the negative cell membrane and offer abundant amine groups for further targeting modification simultaneously. PEI can also display a unique “proton sponge effect” when it is applied to drug delivery [14,15].

Borneol (BO), a commonly used traditional Chinese medicine, is regarded as a promising candidate to promote the absorption of some drugs, especially in oral administration [2,16,17]. Studies showed that BO could enhance the intestinal absorption of some drugs by inhibiting the function of P-gp on the membrane [2,16,17,18]. P-gp is expressed in various kinds of tissues, including intestinal brush border membranes and Caco-2 cells. Known as a model of intestinal epithelial cells, Caco-2 cells are frequently used to estimate drug permeability and substrate activity for efflux transport proteins such as P-gp [19]. Folic acid (FA) is an essential vitamin in cell proliferation. Without nephrotoxicity to the body and kidney retention, folate-modified nanoparticles have been studied to explore the mechanism of FA receptors overexpressed on the surface of intestinal epithelial cells [20,21,22]. The results showed that folate-receptor-mediated endocytosis was one of the major pathways of nanoparticle transmembrane entry into cells [23,24]. Proton-coupled folate transporter (PCFT) is the youngest member of the folate-specific transporters, which is highly expressed in the apical brush border and basolateral membrane of intestinal epithelial cells, affecting intestinal folate absorption [25,26]. The mechanism by which PCFT affects folate absorption is related to proton gradient H^+^. The pH value of the microenvironment in the proximal small intestine ranges between 5.8–6.0, indicating that proton gradients H^+^ are present outside and inside the intestinal cells [27]. With the increase of the proton gradient H^+^ on both sides of the cell membrane, more FA is ingested [28]. However, how PCFT is associated with the absorption of folate-modified nanoparticles has not yet been studied.

In the present study, DTX-loaded PLGA-NPs were prepared, modified with PEI coupling FA (FA-PEI) or BO (BO-PEI) (FA/BO-PLGA-NPs), and the mechanism of enhancing oral absorption was investigated. The characteristics of the designed FA/BO-PLGA-NPs were as follows: (i) drug release from FA/BO-PLGA-NPs and intestinal absorption of nanoparticles, (ii) cell uptake of FA/BO-PLGA-NPs by confocal laser scanning microscopy (CLSM) and BD FACS caliber flow cytometer, and (iii) the relative bioavailability and cytotoxicity or irritation of FA/BO-PLGA-NPs compared with DTX suspension was also studied in rats.

## 2. Materials and Methods 

### 2.1. Materials

DTX was purchased from Biocompounds Pharmaceutical Inc. (Shanghai, China). PLGA (Mw = 15 kDa) was purchased from Jinan Daigang Biological Tech Co., Ltd. Borneol was purchased from Lei Yunshang Drug Store. PEI (Mw = 1.8kDa), Folic acid (USP grade), 1-ethyl-3-(3-dimethylamino propyl) carbodiimide hydrochloride (EDC.HCl, purity > 98%), N-hydroxysuccinimide (NHS, purity > 98%), succinic anhydride (SA, purity > 98%), 4-(dimethylamino) pyridine (DMAP) were provided by Aladdin Chemistry (Shanghai, China). Coumarin-6 was obtained from Sigma-Aldrich (St. Louis, MO, USA). Rhodamine123 (R123) (purity ≥ 95%) was obtained from Wako Pure Chemical Industries, Ltd. Dichloromethane was obtained from Sinopharm Chemical Reagent Co., Ltd. (Shanghai, China). Dimethyl sulfoxide (DMSO), triethylamine (TEA), *N*,*N*-Dimethylformamide (DMF) and other solvents were analytical grade reagents and obtained from Shanghai Lingfeng Chemical Reagent Co., Ltd. Fetal bovine serum (FBS), Dulbecco’s phosphate buffer saline (PBS), Trypsin 0.25%-EDTA and culture medium DMEM were purchased from Biosera (Biosun Science & Technology Co., Ltd., Shanghai, China). Caco-2 cell lines (Caco-2) were provided by Cell Bank of Chinese Academy of Sciences (Shanghai, China).

### 2.2. Synthesis and Characterization of FA-PEI Copolymer

FA-PEI copolymer was synthesized according to a previous study [29]. PEI (258.0 mg, 1.8 kDa) was dissolved in 10 mL of dimethyl sulfoxide (DMSO), and 1 mL of triethylamine (TEA) was added to obtain the PEI solution. Next, 66.2 mg of FA, 69.1 mg of NHS and 115.0 mg of EDC were dissolved in 5 mL of DMSO, and the mixture was stirred in the dark for 4 h to obtain the activated FA solution. FA solution was added dropwise to the PEI solution, and the mixture was stirred in the dark at room temperature under the protection of N_2_ for 48 h. The liquid reaction product was precipitated using acetone (reaction liquid: acetone = 1:15). After centrifugation, a solid precipitate was obtained and purified thrice using acetone. The solid product was dried under vacuum for 24 h and dissolved in water. The obtained product was adjusted to pH 4.5 with 0.2% (*w*/*w*) hydrochloric acid and centrifuged (8000 rpm, 5 min) to remove the precipitate. Finally, after freeze-drying for 72 h, FA-PEI was obtained. The chemical structure of this copolymer was confirmed by ^1^H-NMR spectroscopy (Agilent 400-MR NMR, Agilent Technologies Co., Palo Alto, CA, USA), and compared with the ^1^H-NMR spectrum of FA-PEI reported in literature [29].

### 2.3. Synthesis and Characterization of BO-PEI Copolymer

A total of 2 g of succinic anhydride (SA) was dissolved in 50 mL of dichloromethane, and 500 mg of BO was added to 4-dimethylaminopyridine and TEA. Then, BO solution was added dropwise into SA solution at room temperature for 48 h in an oil bath, and dichloromethane was removed. The mixture product was obtained and stored at 4 °C. A total of 113 mg of EDC with 69 mg of NHS was dissolved in 3 mL of PBS (pH = 5.7) and then gradually added dropwise to 30 mg of the mixture product dissolved in 2 mL of *N*,*N*-dimethylformamide (DMF). Activation reaction was conducted at room temperature after 2 h. After extraction with N-hexane for several times, BO–SA ester (BO–SA) was obtained in the lower layer [30]. BO–SA was dissolved in 2 mL of DMF. Then, 10 mg of PEI (1.8 kDa) was dissolved in 2 mL of DMSO, and then PEI was added dropwise to the BO–SA solution and allowed to react at room temperature overnight. After reaction, the reaction liquid was transferred into a dialysis bag (Mr = 1400). After 24 h of dialysis with ultrapure water and 48 h of lyophilization, BO-PEI was obtained. The chemical structure of this copolymer was confirmed by ^1^H-NMR spectroscopy and FTIR (Nicolet 6700, Thermo Electron Co., Waltham, MA, USA).

### 2.4. Preparation and Characterization of FA/BO-PLGA-NPs

FA/BO-PLGA-NPs were prepared by emulsion solvent evaporation [31]. In brief, 20 mg of PLGA and 1 mg of DTX were dissolved in 1 mL of dichloromethane as the oil phase. Twenty milliliters of PVA (0.1%) solution was prepared as the aqueous phase. The oil phase was dropped into the aqueous phase in ice bath by ultrasonication (power 15%, 5 min). Then, the organic solvent was removed by vapor compression to solidify the PLGA-NPs. BO-PEI and FA-PEI samples were added to ultrapure water solution to obtain BO-PEI (3 mg/mL) and FA-PEI (1 mg/mL). Then, 10 mL of BO-PEI and 10 mL of FA-PEI were mixed together to obtain BO-PEI (1.5 mg/mL) and FA-PEI (0.5 mg/mL). PLGA nanoparticles were dripped slowly into 20 mL of the mixed solution mentioned above to obtain the A milky lucent transparent nanoparticle solution by electrostatic adsorption. The nanoparticles were centrifuged (4 °C, 2000 rpm, 30 min), precipitates were discarded, the supernatant solution was centrifuged (4 °C, 12000 rpm, 30 min), the supernatant was discarded, and the precipitate was washed one or two times with ultrapure water to obtain water solution nanoparticles. Finally, FA/BO-PLGA-NPs were obtained via lyophilization. FA-PLGA-NPs and BO-PLGA-NPs were prepared by the same method. 

Particle sizes and zeta potentials were measured by Zetasizer-ZS90 (Malvern Instruments, Malvern, UK), and morphological characteristics were examined under a transmission electron microscope (TEM, Tecnai G2 spirit Biotwin, FEI, Hillsborough, OR, USA). Encapsulation efficiency (EE%) and drug loading (DL%) were determined as previously described [32]. Briefly, 400 mL of FA/BO-PLGA-NP solution was subjected to ultrafiltration centrifugation (MWCO (molecular weight cut-offs) = 3500 Da, Millipore, Billerica, MA, USA) and centrifuged (4000 rpm, 30 min). The filtrate was injected into HPLC (SPD-20A, Shimadzu, Tokyo, Japan) to detect the amount of free drug (W*_free_*). The total amount of drug (W*_total_*) in FA/BO-PLGA-NPs was extracted using methanol. The lyophilized FA/BO-PLGA-NPs were weighed as (W*_NPs_*). The formulas for encapsulation efficiency (EE%) and drug loading (DL%) were as follows:

EE% = (W*_total_* − W*_free_*)/W*_total_* × 100%, (1)

DL% = (W*_total_* − W*_free_*)/W*_NPs_* × 100%, (2)

where W*_total_* represents the total weight of the drug, W*_free_* represents the weight of the free drug, and W*_NPs_* represents the weight of NPs. Both EE% and DL% were measured by HPLC [29,30]. 

### 2.5. Stability and In Vitro Release of FA/BO-PLGA-NPs

The FA/BO-PLGA-NPs solution was stored at 4 °C and 25 °C, and the size of the nanoparticles was detected periodically within 30 days. The stability of nanoparticles was evaluated according to particle sizes. The in vitro release of FA/BO-PLGA-NPs (pH = 7.4) was studied by dialysis bag diffusion technique. FA/BO-PLGA-NPs and DTX were placed in a dialysis membrane bag (Mw = 3500 Da), and all samples were in a sink condition with the same amount of drug. Then, each group of samples was immersed in 50 mL of phosphate buffer solution (pH = 7.4) in a thermoshaker bath system. The system was maintained at 37 °C and shaken at 100 rpm. After a particular time interval, 1 mL of the dissolution medium was withdrawn and replaced with 1 mL of fresh dissolution medium. The released DTX in the dissolution media was determined by HPLC. Measurements of each batch were performed in triplicates.

### 2.6. HPLC Analytical Method

The amount of DTX loaded into the nanoparticles was measured by HPLC. In brief, the HPLC condition was as follows: column (C_18_, 200 mm × 4.6 mm, Dikma Technologies, Beijing, China), mobile phase (water: acetonitrile = 52:48, volume/volume), and the flow rate was 1.0 mL/min. The column temperature was maintained at 25 °C, the wavelength of ultraviolet-visible detection was set at 230 nm, and the injection volume was 20 µL. All data were analyzed using LC solution software of chromatography system composed of an LC-20AT binary pump and an SPD-20A Ultraviolet-Visible Detector (Shimadzu Corporation, Tokyo, Japan). 

### 2.7. Intestinal Absorption

Male Sprague–Dawley (SD) rats (250 ± 20 g) were fasted for 24 h before the experiment. All animals used in the whole study were treated according to the ethical guidelines of the School of Pharmacy, Shanghai Jiao Tong University. After being anesthetized, the abdominal cavities of the rats were opened, and their intestines were removed. The small intestines (10 cm) were excised quickly, and the underlying mesenteria were removed. Then, the intestines were rinsed and flushed thoroughly with K-R buffer solution. Samples (1 mL) were injected into the intestinal sac, and then the intestines were tied at their two ends. The sample solutions were DTX, PLGA-NPs, FA-PLGA-NPs, BO-PLGA-NPs, and FA/BO-PLGA-NPs. Afterwards, the intestines were placed into K-R buffer nutrition (37 °C, 10 mL) with continuous ventilation. The initial concentrations of all the samples in the small intestine were 20 µg/mL. The samples were withdrawn in 5, 15, 30, 45, 60, 90, and 120 min and were replaced with K-R buffer nutrient solution with the same volume. Afterwards, the samples were centrifuged (4 °C, 2000 rpm, 30 min), and DTX was determined by HPLC. The apparent permeability coefficients (*P_app_*) of DTX were calculated from the slope of the linear portion of the permeability–time profiles using the relationship:

*P_app_* = d*Q_t_*/d*_t_*(1/*AC*_0_), (3)

*R* = *P_app_*(sample)/*P_app_*(control), (4)

where *P_app_* represents the apparent permeability coefficients, *Q_t_* represents the amount of drug in the receptor side, *A* represents the diffusion area, *C*_0_ represents the initial concentration of drug on the donor side, and *R* represents the absorption promoting ratio.

### 2.8. Cell Culture

Caco-2 cells were cultured in DMEM with 20% fetal bovine serum (FBS, Biosera), 1% streptomycin sulfate and penicillin G sodium, and 1% MEM non-essential amino acids. Then cells were maintained in an incubator (37 °C, 5% CO_2_).

### 2.9. Cell Cytotoxicity Assay

The cell cytotoxicity of different nanoparticles was analyzed by 3-(4,5-dimethyl-2-thiazolyl)-2,5-diphenyl-2-*H*-tetrazolium bromide (MTT) assay. Caco-2 cells were seeded into 96-well plates (5 × 10^3^ cells/mL, 100 μL) and cultivated in DMEM at 37 °C in 5% CO_2_ atmosphere for 24 h. Afterwards, the medium was removed and 100 μL of DTX, PLGA-NPs, FA-PLGA-NPs, BO-PLGA-NPs and FA/BO-PLGA-NPs were added to culture for another 48 h. In addition, each DTX, PLGA-NP, FA-PLGA-NP, BO-PLGA-NP and FA/BO-PLGA-NP group containing 0.1, 1.0, 2.5, 10.0 and 25.0 µg/mL of DTX was tested on Caco-2 cells. At last, 20 μL of MTT (5 mg/mL) PBS solution was added into each well and incubated cells for another 4 h. Then, 0.3, 3.0, 30.0, 300.0, and 3000.0 µg/mL of blank FA/BO-PLGA-NPs tested on Caco-2 cells were carried out by the same way. Absorbance was obtained with a microplate reader (Spectra MAX M3, Molecular Devices Corporation, Sunnyvale, CA, USA) at 570 nm. IC_50_ was calculated using GraphPad Prism.

### 2.10. Cellular Uptake Mechanism Studies of Bifunctional Nanoparticles In Vitro

A BD FACS calibur flow cytometer (BD Biosciences) and CLSM were used to study the mechanism of cell uptake. R123 was used as a fluorescent marker to study the cell uptake of BO, and C6 was applied as a fluorescent marker to study the cell uptake of FA. Briefly, free fluorescence dye (R123 or C6), R123-loaded nanoparticles (R123-loaded PLGA-NPs, BO-PLGA-NPs, and FA/BO-PLGA-NPs) and C6-loaded nanoparticles (C6-loaded PLGA-NPs, FA-PLGA-NPs, BO-PLGA-NPs, and FA/BO-PLGA-NPs) were prepared with the same method mentioned in Section 2.4; the final R123 or C6 concentration of each sample was 300 ng/mL. Then, the dye solutions and nanoparticle suspensions mentioned above were incubated with Caco-2 cells (5 × 10^5^) cultured beforehand in 6-well plates at 37 °C overnight. After 4 h, the cells were washed three times with cold PBS, fixed with 4% paraformaldehyde, stained with 4′,6-diamidino-2-phenylindole and analyzed with CLSM (Leica TCS SP5, Wetzlar, Germany). Furthermore, to investigate whether R123 is a specific substrate for efflux pump P-gp, verapamil+R123 (Ver+R123) was set as a positive control group with the concentration of verapamil at 0.3 mM [2], and the cell uptake test was performed as above. In addition, for further determination whether the FA receptor has an effect on the cellular uptake properties of nanoparticles, C6-loaded FA/BO-PLGA-NPs were added to Caco-2 cells pre-incubated with 2 mL of FA (1 mM) (FA/BO-PLGA-NPs+FA) for 1 h [33]. 

For quantitative investigation of the cellular uptake of nanoparticles, flow cytometry was introduced. Caco-2 cells (5 × 10^5^) were seeded into six-well plates. After 24 h, cells were incubated with different nanoparticles mentioned above for another 4 h of incubation. Then, the cells were washed with PBS and analyzed using flow cytometer.

Moreover, to further understand whether the inhibition or up-regulation of PCFT affects the cellular uptake of nanoparticles, carbonylcyanide *p*-trifluoromethoxyphenylhydrazone (FCCP), an inhibitor of PCFT, and vitamin D3 were introduced. In brief, caco-2 cells were treated with C6-loaded FA/BO-PLGA-NPs, which were also added with or without FCCP (FA/BO-PLGA-NPs, FA/BO-PLGA-NPs+FCCP) [34]. Meanwhile, the effect of vitamin D3 on the cell uptake of FA/BO-PLGA-NPs via PCFT transporter was also studied. Vitamin D3, with concentrations of 0, 50, 250, and 500 nM, was added to caco-2 cells (5 × 10^5^) that were incubated beforehand for two days and then cultured for another three days [35]. Afterwards, the C6-loaded FA/BO-PLGA-NPs were added to cell cultures pre-treated with different concentrations of vitamin D3. CLSM and flow cytometry analysis were performed using the same methods mentioned above.

### 2.11. Pharmacokinetics Study

Pharmacokinetics study was carried out in male SD rats (230 ± 10 g) that were bought from Shanghai Jiao Tong University (SJTU) Laboratory Animal Center (Shanghai, China). Male SD rats (230 ± 10 g) were randomly divided into four groups and each animal was fasted with free access to water. Then they were administrated orally with DTX suspension, PLGA-NPs, BO-PLGA-NPs and FA/BO-PLGA-NPs; the DTX concentration in each group was 10 mg/kg. Then, each sample was collected beforehand from retro-orbital plexus at 5, 15, 30, 45, 60, 90, 120, 240, 360, 480 and 720 min and placed into microcentrifuge tubes with heparin. Then, the samples were centrifugated at 6000 rpm for 6 min and acetonitrile was added. After centrifugation (12,000 rpm, 6 min), the upper supernatant was filtered and transferred into another tube. A total of 20 μL of sample was aspirated and injected into the HPLC. The DTX pharmacokinetic parameters were analyzed using DAS software. 

### 2.12. Intestinal Irritation of FA/BO-PLGA-NPs

The nine male SD rats (230 ± 10 g) from Shanghai Jiao Tong University (SJTU) Laboratory Animal Center (Shanghai, China) were assigned to three groups at random and each animal was fasted. All rats had free access to water before oral administration. The group treated with no drug was the control group. The other two groups were treated with DTX suspension and FA/BO-PLGA-NPs, and the DTX concentration of each group was 10 mg/kg. The intestines were removed and fixed in 4% paraformaldehyde at room temperature for 12 h. The abdominal cavity was opened and the jejunum and ileum sections were removed and fixed in 4% paraformaldehyde. After fixation, all samples were dehydrated with ethanol and cleared in xylene. Then, the samples were infiltrated and embedded in paraffin, and microtome sections were cut from the paraffin-embedded blocks. Sections of jejunum and ileum were stained with hematoxylin and eosin prior to microscopic examination [36]. The stained slides were examined with Leica microscope, and the morphological changes were assessed.

### 2.13. Statistical Analysis

All results were presented as mean ± standard deviation (SD). All experiments were repeated at least three times. Statistical analysis was conducted using Student’s *t*-test. * p < 0.05 reflects significant difference, and ** *p* < 0.01 reflects extremely significant difference.

## 3. Results and Discussion

### 3.1. Synthesis and Characterization of FA-PEI Copolymer

**Figure 1:**
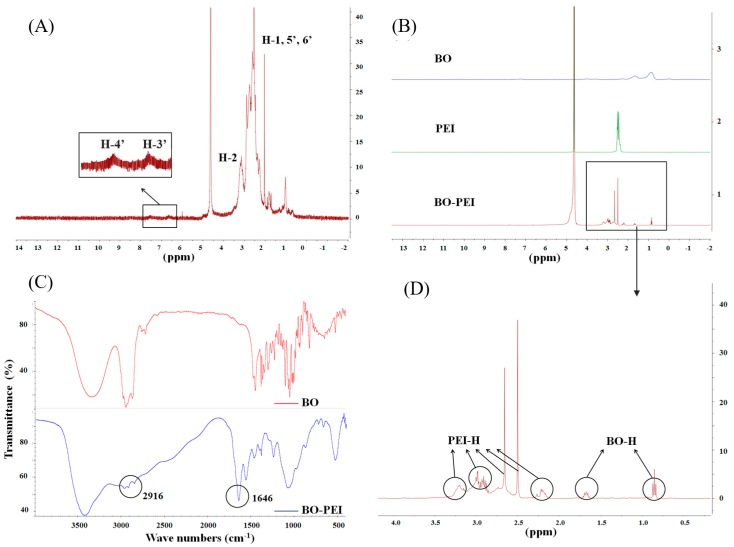
(**A**) Characterization of polyethyleneimine–folic acid (FA-PEI) copolymer by ^1^H-NMR; (**B**,**D**) characterization of polyethyleneimine–borneol (BO-PEI) copolymer through ^1^H-NMR spectroscopy; (**C**) characterization of BO-PEI copolymer through FTIR.

Figure 1A shows the ^1^H-NMR spectra of FA-PEI and the major proton signals. H-1 (δ = 2.3–2.9 ppm) indicated that the proton signals of the PEI chain were not connected with the amide bond, while H-2 (δ = 3.3 ppm) indicated connection with the amide bond. H-3′ (δ = 6.5 ppm) and H-4′ (δ = 7.5 ppm) are the proton peaks of benzene ring on FA, which was nearly the same as that of the reference [29]. After ^1^H-NMR analysis and comparison with the reference, we could conclude that FA was conjugated with PEI successfully. However, the signals of FA were relatively weak, which might connect with the low concentration of FA in the polymer. 

### 3.2. Synthesis and Characterization of BO-PEI Copolymer

The ^1^H-NMR spectra of BO, PEI, BO-PEI, and BO were compared and successfully conjugated to PEI (Figure 1B). The ^1^H-NMR spectrum of BO-PEI was further analyzed in Figure 1D. The proton peaks (δ = 0.8–1.2 ppm) belonged to three methyl nuclei (–C–(CH_3_)_2_–C–(CH_3_)–C–O) of the BO. The proton peaks associated with the PEI were found at δ = 2.2 ppm (–NCH_2_CH_2_CO–), δ = 2.5 ppm (–CONHCH_2_CH_2_N–), δ = 2.7 ppm (–CONHCH_2_CH_2_N–), δ = 2.9 ppm (–NCH_2_CH_2_CO–), δ = 3.1 ppm (–CONHCH_2_CH_2_NH_2_), and δ = 3.2 ppm (–NCH_2_CH_2_NHCO–). The IR spectra of BO and BO-PEI are shown in Figure 1C. The peaks at 1646 cm^−1^ were the characteristic peaks of an amide bond (ν_C=O_), and that at 2916 cm^−1^ was the characteristic absorption peak of BO nucleus after bonding to the surface of PEI in the IR spectrum of BO-PEI. The absorption peak at 1115 cm^−1^ appeared after ν_C–O–C_ ester skeleton vibration. After ^1^H-NMR mapping and FTIR analysis, we could confirm that BO-PEI was synthesized successfully. 

### 3.3. Characterization of FA/BO-PLGA-NPs 

**Figure 2:**
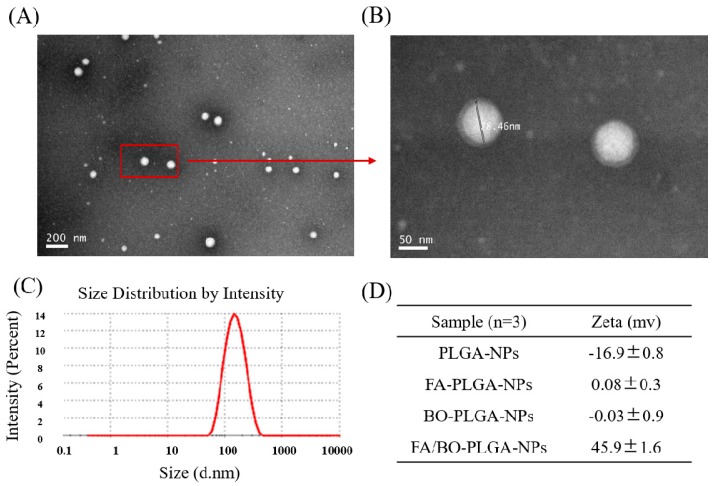
Characterization of FA/BO-polylactic-co-glycolic acid (PLGA)-NPs. TEM images of FA/BO-PLGA-NPs (**A**: 200 nm and **B**: 50 nm), and size was 78.46 nm in (**B**: left); (**C**): size distribution of FA/BO-PLGA-NPs; (**D**): zeta potential of PLGA-NPs, FA-PLGA-NPs, BO-PLGA-NPs, FA/BO-PLGA-NPs.

The TEM photographs show that FA/BO-PLGA-NPs are spherical in shape, obvious in structure, and have no aggregation (Figure 2). After amplification, the halo could clearly be seen around the surface layer of nanoparticles. Obviously, the halo should be the polymer of FA-PEI and BO-PEI, suggesting that FA-PEI and BO-PEI were well adsorbed on the PLGA-nanoparticles (Figure 2B). The average size of final nanoparticles was 137.0 ± 2.1 nm (Figure 2C). Zeta potential is related to the stability of particle dispersion, which represents the strength of repulsion or attraction between nanoparticles. PLGA-NPs were negatively charged, and the zeta potential was −16.9 ± 0.8 mV (Figure 2D). After FA-PEI (0.5 mg/mL) or BO-PEI (0.5 mg/mL) electrostatic adsorption, the zeta potential of FA-PLGA-NPs and BO-PLGA-NPs were 0.08 ± 0.3 and −0.03 ± 0.9 mV, respectively. The –NH exposed outside the PEI branch chain caused the positive charge of the nanoparticles. Owing to FA-PEI (0.5 mg/mL) and BO-PEI (1.5 mg/mL) modified PLGA-NPs, nanoparticles became positively charged. The zeta potential of nanoparticles was 45.9 ± 1.6 mV, which was larger for higher PEI concentrations and represented nanoparticles with good stability [37]. The EE% and DL% were 80.3 ± 1.8% and 2.3 ± 0.3%, respectively.

### 3.4. Stability and In Vitro Release of FA/BO-PLGA-NPs

**Figure 3:**
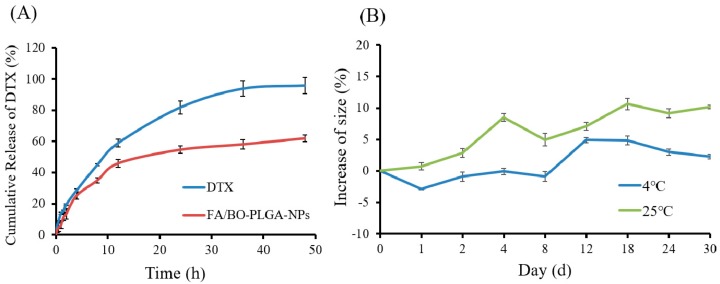
(**A**): Cumulative release of DTX in FA/BO-PLGA-NPs; (**B**): stability of FA/BO-PLGA-NPs: size change of nanoparticles within 30 days.

The in vitro release study of FA/BO-PLGA-NPs (pH = 7.4) was carried out by the dialysis bag diffusion technique. Tween-80 (0.1%) was added to the release medium to improve the release of the drug. As shown in Figure 3A, the cumulative release of DTX in FA/BO-PLGA-NPs reached 60% at 48 h without abrupt release, the release of which was slower than DTX suspension. The increased rate size of FA/BO-PLGA-NPs compared to the initial value was shown in Figure 3B. In contrast, the particle size of nanoparticles was relatively stable at 4 °C for 30 days, whereas this increased obviously at 25 °C. Therefore, FA/BO-PLGA-NPs were stored at 4 °C. 

### 3.5. Intestinal Absorption

**Figure 4:**
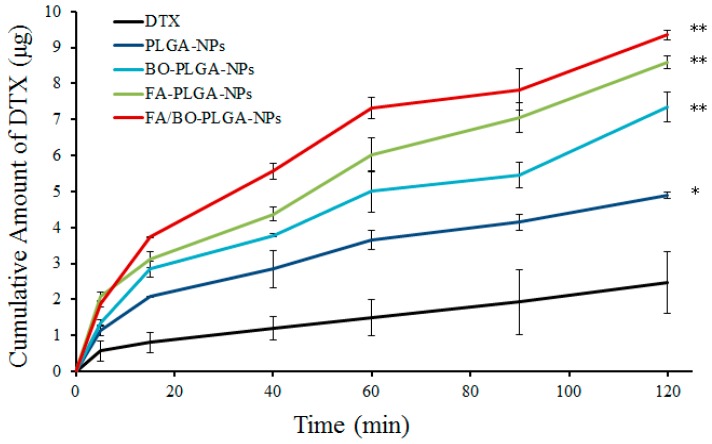
Small intestinal absorption in vitro: cumulative amount of DTX in 2 h. * *p* < 0.05, ** *p* < 0.01.

The in vitro transports of DTX from different kinds of nanoparticles were studied using rat gut sacs. The ultimate cumulative amount of DTX across the small intestine is shown in Figure 4. From Figure 4, the order of small intestinal absorption was as follows: DTX < PLGA-NPs < BO-PLGA-NPs < FA-PLGA-NPs < FA/BO-PLGA-NPs. The amount of absorbed DTX of FA/BO-PLGA-NPs reached the highest in 2 h among all groups. The absorption of DTX in DTX suspension, PLGA-NPs, FA-PLGA-NPs, BO-PLGA-NPs, and FA/BO-PLGA-NPs were 2.5 ± 0.9, 4.9 ± 0.1, 8.6 ± 0.2, 7.3 ± 0.4, and 9.3 ± 0.1 µg, respectively. Nanoparticles can significantly improve the intestinal absorption of DTX (*p* < 0.05), and FA/BO-PLGA-NPs exhibited the strongest absorption. The permeability coefficients (*P_app_*) and the absorption enhancement ratios (*R*) of DTX are displayed in Table 1. FA/BO-PLGA-NPs produced a significantly higher *P_app_*, which exhibited a 6.0-fold increase compared with the DTX suspension. The low absorption of DTX can be attributed to its transportation from the intestinal cells into the lumen by a P-gp-mediated efflux system [38]; BO’s inhibition of the function of P-gp and the FA receptor’s targeting of cells enhanced the penetration of FA/BO-PLGA-NPs into the intestinal epithelial cells in the gastrointestinal tract, thereby promoting the intestinal absorption of DTX in vitro [18,21]. 

### 3.6. Cell Cytotoxicity Assay

**Figure 5:**
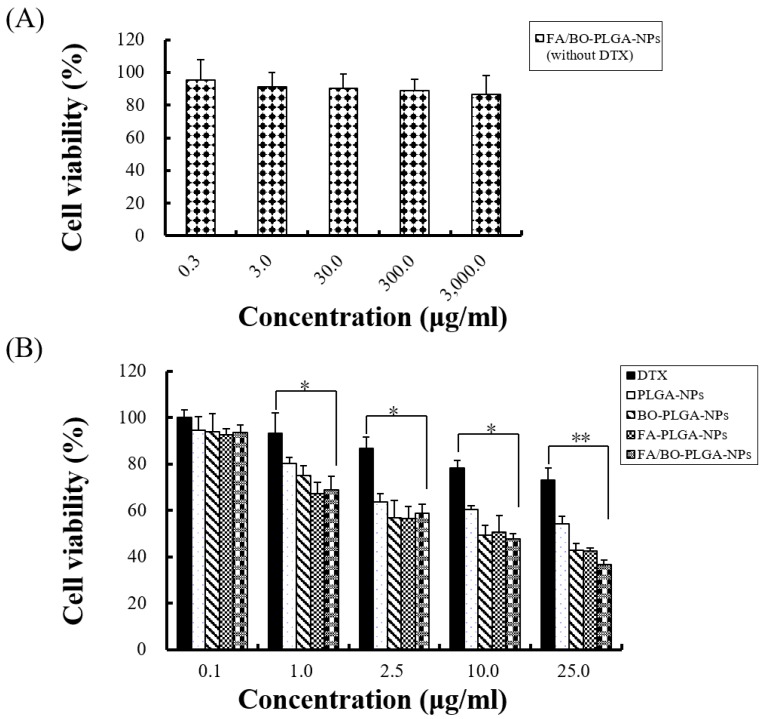
Cell cytotoxicity assay of different nanoparticles was evaluated by MTT assay. (**A**): empty FA/BO-PLGA-NPs without DTX. * *p* < 0.05, ** *p* < 0.01. (**B**): various concentrations of DTX, PLGA-NPs, FA-PLGA-NPs, BO-PLGA-NPs and FA/BO-PLGA-NPs.

In the MTT part of the study, Caco-2 cells were used to evaluate the cytotoxicity of nanoparticles and blank FA/BO-PLGA-NPs with various drug concentrations. In Figure 5A, the result showed that blank FA/BO-PLGA-NPs caused little cytotoxicity on Caco-2 cells because the viability of cells was more than 90% in each concentration. In Figure 5B, the cell viability decreased with the increasing concentration of DTX in diverse kinds of nanoparticles. Compared with the control group (DTX), the inhibition rate of Caco-2 cells was higher in the nanoparticles containing the same concentration of DTX. The results of the MTT assay, shown in Table 2, also indicated that DTX in different formulations inhibited Caco-2 cells. In this experiment, the IC_50_ of FA/BO-PLGA-NPs was the lowest, which may be related to the passive targeting of nanoparticles, the active targeting of FA, and the effect on inhibiting the function of P-gp by BO [2,25]. The MTT data provided referential information for our further cell experiments.

### 3.7. Cellular Uptake Mechanism Studies of Bifunctional Nanoparticles

**Figure 6:**
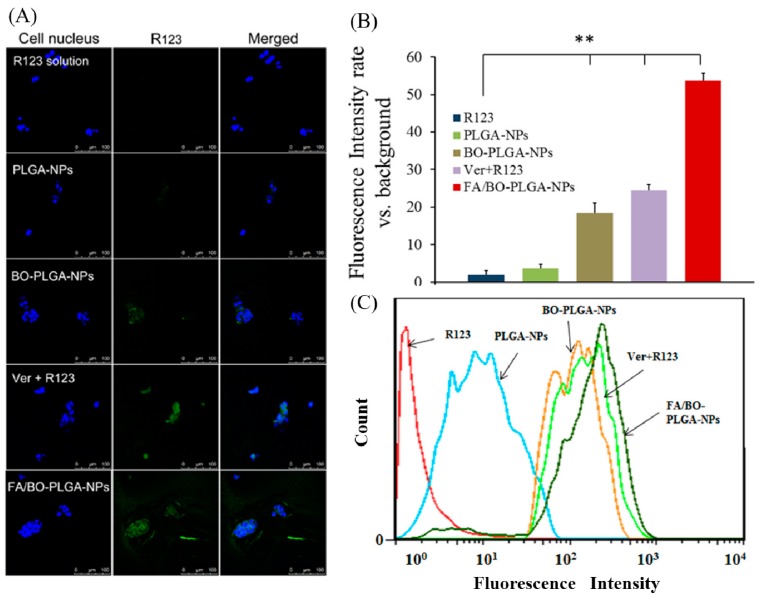
Confocal laser scanning microscopy (CLSM) images (**A**) and fluorescence quantitative analysis (**B**) of Caco-2 cells after incubation with R123, PLGA-NPs, BO-PLGA-NPs, FA/BO-PLGA-NPs, and the group of verapamil+R123 (Ver+R123) was set as the positive control group, with the concentration of verapamil being 0.3 mM (R123 concentration of 300 ng/mL) for 4 h. Columns from left to right correspond to DAPI, R123 and an overlay of DAPI and R123, respectively. Flow cytometry results (**C**) of Caco-2 cells treated with R123, PLGA-NPs, BO-PLGA-NPs, FA/BO-PLGA-NPs and the group of verapamil+R123 (Ver+R123) was set as the positive control group, with the concentration of verapamil being 0.3 mM (R123 concentration of 300 ng/mL) for 4 h. * *p* < 0.05, ** *p* < 0.01.

R123 is a specific substrate for efflux pump P-gp. Thus, R123 was selected to visualize the cellular uptake of nanoparticles instead of DTX. Verapamil is extensively characterized as a P-gp inhibitor to increase oral bioavailability [19,39]. In addition, our previous work and some reports have showed that BO was effective in inhibiting the P-gp-mediated efflux pump [2,40,41]. As shown in Figure 6, compared with R123, the fluorescence intensity of Ver+R123 increased markedly. As can be seen intuitively, fluorescence uptake in BO-PLGA-NPs was similar to that of Ver+R123, which was consistent with previous studies [2,42]. Compared with PLGA-NAs, the accumulation of R123 obviously increased in the presence of BO, which further confirmed that BO has an effect on inhibiting the efflux of P-gp. After further modification of FA-PEI and BO-PEI, the accumulation of R123 in FA/BO-PLGA-NPs was the highest among all groups. The order of fluorescence uptake was as follows: FA/BO-PLGA-NPs > Ver+R123 > BO-PLGA-NPs > PLGA-NPs > R123. In addition to the effect of BO on the uptake mechanism of FA/BO-PLGA-NPs, FA receptor may also be related [23,24]. 

**Figure 7:**
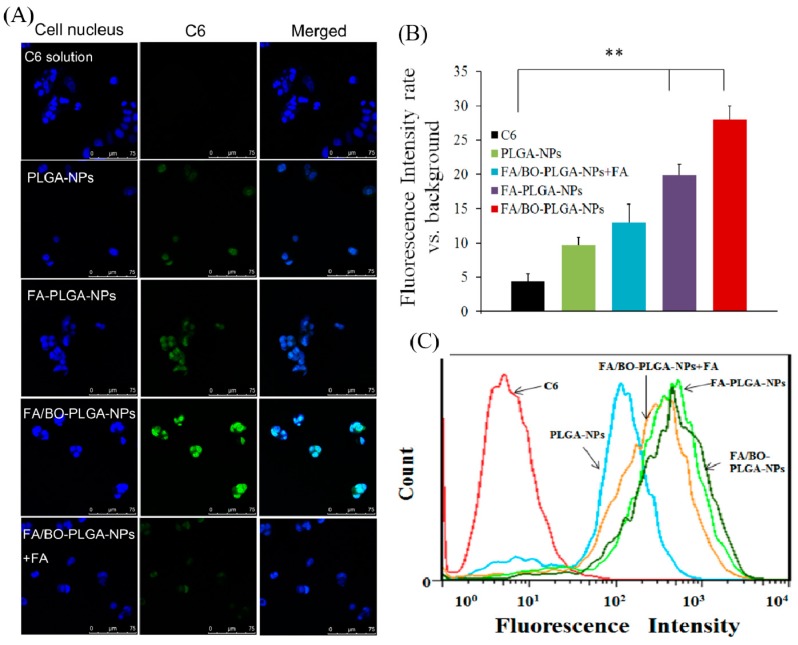
CLSM images (**A**) and fluorescence quantitative analysis (**B**) of Caco-2 cells after incubation with coumarin-6, PLGA-NPs, FA-PLGA-NPs, FA/BO-PLGA-NPs and FA/BO-PLGA-NPs with 2 mL FA (1 mM) for pre-incubated cells (coumarin-6 concentration of 300 ng/mL) for 4 h. Columns from left to right correspond to DAPI, coumarin-6 and an overlay of DAPI and coumarin-6, respectively. Flow cytometry results (**C**) of Caco-2 cells treated with coumarin-6, PLGA-NPs, FA-PLGA-NPs, FA/BO-PLGA-NPs and FA/BO-PLGA-NPs with 2 mL FA (1 mM) for pre-incubated cells (coumarin-6 concentration of 300 ng/mL) for 4 h. * *p* < 0.05, ** *p* < 0.01.

C6 was applied as a fluorescent marker to study the cell uptake of FA. The order of uptake of each group was as follows: FA/BO-PLGA-NPs > FA-PLGA-NPs > PLGA-NPs > C6 (Figure 7). A significant difference was observed between the uptake of FA-PLGA-NPs and C6 (*p* < 0.01), which was related to the endocytosis mediated by FA receptors. Studies have shown that FA receptor-mediated endocytosis helped nanoparticles transmembrane into cells [23,24]. With the addition of FA to Caco-2 cell for pre-incubation and FA/BO-PLGA-NPs, the uptake of C6 was found to be significantly reduced, which further confirmed the role of FA receptor [31]. In addition, the uptake of PLGA-NPs was greater than that of C6, which was related to the endocytosis of PLGA into cells [43]. 

**Figure 8:**
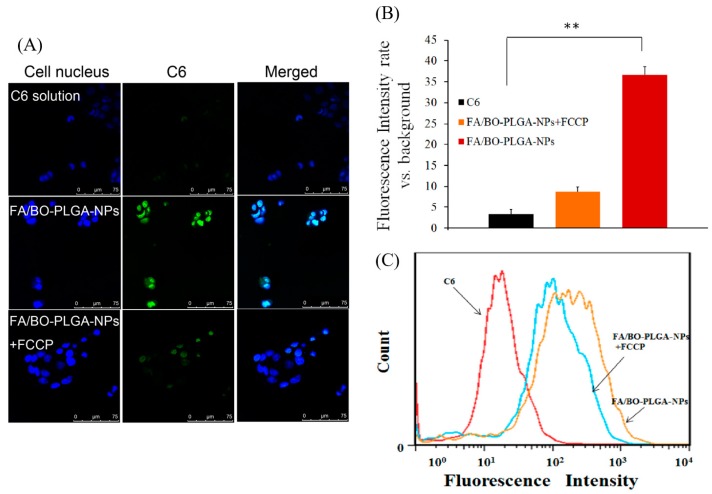
CLSM images (**A**) and fluorescence quantitative analysis (**B**) of Caco-2 cells after incubation with coumarin-6, FA/BO-PLGA-NPs and FA/BO-PLGA-NPs with carbonylcyanide *p*-trifluoromethoxyphenylhydrazone (FCCP) (1 mM) (coumarin-6 concentration of 300 ng/mL) for 4 h. Columns from left to right correspond to DAPI, coumarin-6 and an overlay of DAPI and coumarin-6, respectively. Flow cytometry results (**C**) of Caco-2 cells treated with coumarin-6, FA/BO-PLGA-NPs and FA/BO-PLGA-NPs with FCCP (1 mM) (coumarin-6 concentration of 300 ng/mL) for 4 h. * *p* < 0.05, ** *p* < 0.01.

The role which PCFT played in the cell uptake of FA-mediated nanoparticles was investigated. As shown in Figure 8B, the order of intake of each group was as follows: FA/BO-PLGA-NPs > FA/BO-PLGA-NPs+FCCP > C6. Qiu added the proton inhibitor FCCP to destroy the proton gradient in the cells, and the amount of FA was significantly decreased [44], which was consistent with the results of FA-mediated nanoparticles in our study. Previous study also found that 3H-folic acid can be inhibited with FCCP [45]. The mechanism of PCFT-affected FA absorption was related to proton gradients H^+^. The pH value in the microenvironment of the proximal small intestine was 5.8–6.0, which indicated that proton gradients H^+^ might be closely related to the absorption of FA-mediated nanoparticles. In this way, it might be speculated that the accumulation of FA/BO-PLGA-NPs was increased by adding the proton inhibitor FCCP to destroy proton gradients H^+^. 

**Figure 9:**
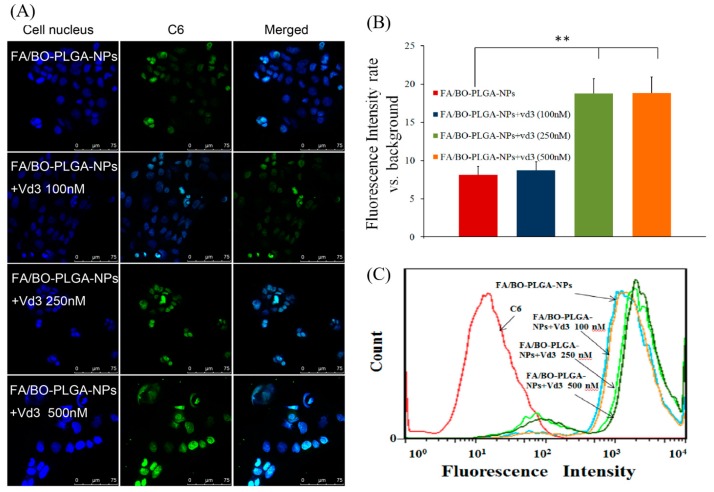
CLSM images (**A**) and fluorescence quantitative analysis (**B**) of Caco-2 cells after incubation with vitamin D3 (vd3) with concentrations of 0 nM, 100 nM, 250 nM and 500 nM applied to the culture cells for three days. Afterwards, we added FA/BO-PLGA-NPs to incubated cells (coumarin-6 concentration of 300 ng/mL) for 4 h. Columns from left to right correspond to DAPI, coumarin-6 and an overlay of DAPI and coumarin-6, respectively. Flow cytometry results (**C**) of Caco-2 cells incubated with vitamin D3 with concentrations of 0 nM, 100 nM, 250 nM and 500 nM applied to culture cells for three days and then treated with FA/BO-PLGA-NPs (coumarin-6 concentration of 300 ng/mL) for 4 h. * *p* < 0.05, ** *p* < 0.01.

The cellular uptake of FA/BO-PLGA-NPs was conducted with vitamin D3 at different concentrations (Figure 9), and the order was as follows: FA/BO-PLGA-NPs+vd3 (500 nM) ≈ FA/BO-PLGA-NPs+vd3 (250 nM) > FA/BO-PLGA-NPs+vd3 (100 nM) > FA/BO-PLGA-NPs. After incubation with vitamin D3 to Caco-2 cells, for the FA/BO-PLGA-NPs+vd3 (250/500 nM) group of nanoparticles, adsorption into cells was greatly improved. This result might be attributed to the fact that the expression levels of endogenous PCFT in Caco-2 cells increased depending on the amount of vitamin D3, which can up-regulate the expression and function of PCFT [35]. Moreover, it could be observed that the cellular uptake was related to the increasing concentration of vitamin D3, suggesting the dose-dependence of the nanoparticles in the absorption. Two-hundred and fifty nanomoles is the optimum concentration of vitamin D3 to induce the expression of PCFT. Meanwhile, the inhibition or up-regulation of PCFT affected the cellular uptake of nanoparticles, which was probably ascribed to the transport of PCFT and FA-mediated nanoparticles. Thus, we can infer that PCFT may be responsible for the transfer of folate-modified nanoparticles. However, further study, such as the knockout of PCFT gene by siRNAs, is essential to verify the hypothesis.

### 3.8. Pharmacokinetics Study

**Figure 10:**
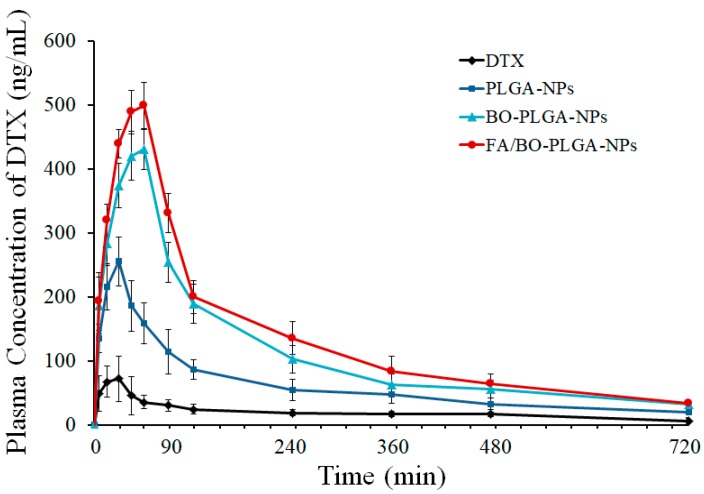
The plasma concentration–time profiles of DTX in SD rats (*n* = 6) after the oral administration of DTX suspension, PLGA-NPs, BO-PLGA-NPs and FA/BO-PLGA-NPs with the dose of 10 mg of DTX/kg to rats, respectively.

The plasma concentration–time curves of different nanoparticles are shown in Figure 10. The order of the peak areas of DTX blood concentration in each group was as follows: FA/BO-PLGA-NPs > BO-PLGA-NPs > PLGA-NPs > DTX. A remarkable increase in the plasma concentrations of DTX was observed in FA/BO-PLGA-NPs. Table 3 shows the pharmacokinetic parameters [*C*_max_, AUC_0–12_, *T*_max_ (h)] of DTX after oral administration. The maximum concentrations (*C*_max_) of PLGA-NPs, BO-PLGA-NPs, and FA/BO-PLGA-NPs were 3.7, 6.1, and 7.1 times higher than that of DTX suspension, respectively. The relative bioavailability of PLGA-NPs, BO-PLGA-NPs, and FA/BO-PLGA-NPs were 2.8, 4.7, and 6.8 times higher than that of DTX suspension, respectively, which suggested a higher capacity of FA/BO-PLGA-NPs in the oral absorption of DTX. *T*_max_ (h) of nanoparticles were longer than DTX, which suggested that the uptake of DTX in the nanoparticles in encapsulated form can be administered through the GI tract for their small particle sizes in absorption [46]. Moreover, combined with the results of cell experiments, the absorption of BO-PLGA-NPs and FA/BO-PLGA-NPs was also due to P-gp inhibition with BO and absorption with FA receptor [2,24]. Furthermore, PCFT, which had high affinity to FA, which was expressed in the apical brush border and basolateral membrane of intestinal epithelial cells, might contribute to promoting the absorption of FA-PLGA-NPs and FA/BO-PLGA-NPs into intestinal cells [27,28]. In Figure 10, we found that the plasma concentration of the control group (DTX suspension) decreased sharply after the peak of concentration while that of nanoparticles decreased gradually. The reason for the slow rate of nanoparticles after administration was that the drug was not easily released from the nanoparticles [47]. DTX inside PLGA nanoparticles may possibly perforate the blood vessel wall and then enter into other tissues or organs. In addition, DTX may be protected with a PLGA shell to avoid being eliminated in the early phase after administration [48]. Therefore, FA/BO-PLGA-NPs played an active role in improving the oral absorption of DTX. 

### 3.9. Intestinal Irritation of FA/BO-PLGA-NPs 

**Figure 11:**
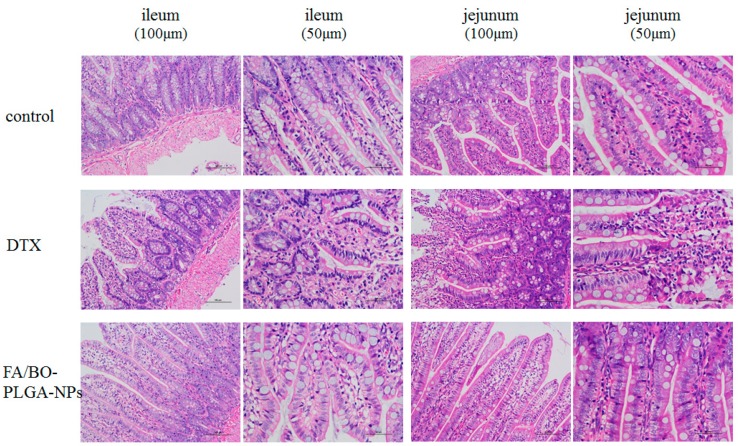
Histological sections of ileum and jejunum in rat’s small intestine with different scales (100 μm and 50 μm) after oral administration of no drug (control group), DTX suspension and FA/BO-PLGA-NPs under haematoxylin–eosin (HE) staining.

Irritations of jejunum and ileum were performed to evaluate whether DTX and FA/BO-PLGA-NPs had obvious stimulus or irritation. As shown in Figure 11, compared with the control group, no obvious changes were observed in the cell tissue of the FA/BO-PLGA-NP group, while the color in DTX group was darker, indicating cell tissue damage. Therefore, FA/BO-PLGA-NPs had no irritation and caused no potential toxic side effects to the rats’ intestines. H&E staining confirmed the safety of FA/BO-PLGA-NPs [49]. Furthermore, FA/BO-PLGA-NPs can enter the circulation of blood as proven in pharmacokinetics study.

## 4. Conclusions

This study developed an active bifunctional nanoparticle formulation (FA/BO-PLGA-NPs) to increase the oral absorption of DTX and explored the mechanism by which nanoparticles transport across intestinal cells. In our study, PEI modified with FA (FA-PEI) or BO (BO-PEI) was synthesized successfully and used to prepare nanoparticles through electrostatic interaction. BO promoted cellular uptake by inhibiting the function of P-gp, and FA was found to enhance the active targeting of nanoparticles. The absorption ability of FA/BO-PLGA-NPs was the strongest in the study of intestinal absorption in vitro and through pharmacokinetics. Therefore, FA/BO-PLGA-NPs can be promising vectors for the oral administration of water-insoluble drugs.

## Figures and Tables

**Table 1:** Small intestinal absorption in vitro: apparent permeability coefficient (*P_app_*) and absorption promoting rate (*R*).

Formulation	*P_app_* (* 10^−6^ cm/s) (Mean ± SD)	Absorption Promoting Ratio (*R*)
DTX	1.4 ± 1.1	1
PLGA-NPs	3.7 ± 0.6 *	2.7 *
FA-PLGA-NPs	7.6 ± 1.9 **	5.5 **
BO-PLGA-NPs	6.9 ± 0.5 **	4.9 **
FA/BO-PLGA-NPs	7.8 ± 3.9 **	6.0 **

* *p* < 0.05, ** *p* < 0.01, compared with docetaxel (DTX) (the control group).

**Table 2:** IC_50_ of the experimental groups on Caco-2 cells.

Groups	IC_50_ (μg/mL) ± SD
DTX	178.20 ± 0.50
PLGA-NPs	28.70 ± 0.21 **
BO-PLGA-NPs	9.66 ± 0.09 **
FA-PLGA-NPs	8.81 ± 0.10 **
FA/BO-PLGA-NPs	6.99 ± 0.08 **

* *p* < 0.05, ** *p* < 0.01, compared with DTX (the control group).

**Table 3:** Pharmacokinetic parameters of DTX in SD rats (*n* = 6) after oral administration of DTX suspension as a control group, and the other groups were treated with PLGA-NPs, BO-PLGA-NPs and FA/BO-PLGA-NPs with the dose of 10 mg of DTX/kg to rats, respectively. (mean ± SD, *n* = 6).

	DTX	PLGA-NPs	BO-PLGA-NPs	FA/BO-PLGA-NPs
*C*_max_ (mg/L)	0.07 ± 0.87	0.26 ± 0.04 **	0.43 ± 1.01 **	0.50 ± 0.53 **
*T*_max_ (h)	0.44 ± 0.00	0.57 ± 0.03	0.92 ± 0.05 **	1.06 ± 0.09 **
AUC_0–12h_ (mg/L*h)	0.23 ± 0.12	0.64 ± 0.14 **	1.07 ± 0.02 **	1.57 ± 0.60 **
F_rel_ (%)	/	278.3	465.2	682.6

* *p* < 0.05, ** *p* < 0.01, compared with DTX (the control group); F_rel_, relative bioavailability.
